# Blood-feeding, susceptibility to infection with Schmallenberg virus and phylogenetics of *Culicoides* (Diptera: Ceratopogonidae) from the United Kingdom

**DOI:** 10.1186/s13071-018-2650-x

**Published:** 2018-02-27

**Authors:** James Barber, Lara E. Harrup, Rhiannon Silk, Eva Veronesi, Simon Gubbins, Katarzyna Bachanek-Bankowska, Simon Carpenter

**Affiliations:** 1 Vector-borne Viral Disease Programme, The Pirbright Institute, Pirbright, Surrey, UK; 2 National Centre for Vector Entomology, Institute of Parasitology, University of Zürich, Winterthurerstr. 266a, 8057 Zürich, Switzerland

**Keywords:** Vector competence, Biting midges, Arbovirus, SBV, Orthobunyavirus, DNA barcode, *cox1*

## Abstract

**Background:**

*Culicoides* biting midges (Diptera: Ceratopogonidae) are responsible for the biological transmission of internationally important arboviruses of livestock. In 2011, a novel *Orthobunyavirus* was discovered in northern Europe causing congenital malformations and abortions in ruminants. From field studies, *Culicoides* were implicated in the transmission of this virus which was subsequently named Schmallenberg virus (SBV), but to date no assessment of susceptibility to infection of field populations under standardised laboratory conditions has been carried out. We assessed the influence of membrane type (chick skin, collagen, Parafilm M®) when offered in conjunction with an artificial blood-feeding system (Hemotek, UK) on field-collected *Culicoides* blood-feeding rates. Susceptibility to infection with SBV following blood-feeding on an SBV-blood suspension provided *via* either (i) the Hemotek system or *via* (ii) a saturated cotton wool pledglet was then compared. Schmallenberg virus susceptibility was defined by RT-qPCR of RNA extractions of head homogenates and related to *Culicoides* species and haplotype identifications based on the DNA barcode region of the mitochondrial cytochrome *c* oxidase 1 (*cox*1) gene.

**Results:**

*Culicoides* blood-feeding rates were low across all membrane types tested (7.5% chick skin, 0.0% for collagen, 4.4% Parafilm M®, with 6029 female *Culicoides* being offered a blood meal in total). Susceptibility to infection with SBV through membrane blood-feeding (8 of 109 individuals tested) and pledglet blood-feeding (1 of 94 individuals tested) was demonstrated for the Obsoletus complex, with both *C. obsoletus* (Meigen) and *C. scoticus* Downes & Kettle susceptible to infection with SBV through oral feeding. Potential evidence of cryptic species within UK populations was found for the Obsoletus complex in phylogenetic analyses of *cox*1 DNA barcodes of 74 individuals assessed from a single field-site.

**Conclusions:**

Methods described in this study provide the means to blood-feed Palaearctic *Culicoides* for vector competence studies and colonisation attempts. Susceptibility to SBV infection was 7.3% for membrane-fed members of the subgenus *Avaritia* and 1.1% for pledglet-fed. Both *C. obsoletus* and *C. scoticus* were confirmed as being susceptible to infection with SBV, with potential evidence of cryptic species within UK Obsoletus complex specimens, however the implications of cryptic diversity in the Obsoletus complex on arbovirus transmission remains unknown.

**Electronic supplementary material:**

The online version of this article (10.1186/s13071-018-2650-x) contains supplementary material, which is available to authorized users.

## Background

In 2011, a novel *Orthobunyavirus*, provisionally named Schmallenberg virus (SBV), was discovered in Germany and was subsequently found to cause foetal abnormalities and abortions in ruminants [1–3]. To date, no convincing route for the incursion of SBV into Europe has been provided, echoing the outbreak of bluetongue virus (BTV) serotype 8 that occurred in the same region 5 years prior to this event [4]. Subsequently, surveys based upon detection of SBV antibodies, viral RNA and clinical disease in livestock demonstrated that the virus spread rapidly across the northwestern region of Europe during 2011–2012 [5–8]. Countries in the region then reported significant reductions in additional clinical cases and seroconversion to SBV infection during 2013–2014 [5, 9], driven by a lack of naïve hosts. Since these studies, recirculation of the virus in northwestern Europe has occurred [10], but the majority of monitoring surveys have been scaled back following reductions in clinical cases of disease and the deployment of an effective vaccine [11].

*Culicoides* biting midges (Diptera: Ceratopogonidae) were suspected as the primary biological vectors of SBV prior to direct study of transmission in the field, due to their involvement in the BTV epidemic in northwestern Europe 5 years earlier [12], and the close phylogenetic relationship of SBV with other *Culicoides*-borne orthobunyaviruses [1, 13]. This hypothesis was confirmed by a series of field-based trials in Belgium, the Netherlands and France that detected significant quantities of SBV RNA in *Culicoides* collected in close proximity to livestock [14–18], while failing to detect virus in mosquitoes [19]. Techniques to assess the probability of field transmission of SBV by *Culicoides* and mosquitoes were standardised using colony lines of *Culicoides sonorensis* Wirth & Jones and *Culicoides nubeculosus* (Meigen) infected using artificial membrane-based techniques [20, 21].

Within northwestern Europe, the *Culicoides* fauna on farms and stables are dominated by species classified within the subgenus *Avaritia* [18, 22, 23]. Until recently four species within this subgenus have been identified within this region and are commonly referred to as the Obsoletus group, despite a lack of monophyly: *Culicoides obsoletus* (Meigen) and *Culicoides scoticus* Downes & Kettle; *Culicoides dewulfi* Goetghebuer and *Culicoides chiopterus* (Meigen) [24–26]. Several additional cryptic species have also recently been proposed whose taxonomic status, prevalence, abundance and involvement in transmission of arboviruses remains uncertain [27–29]. To date, these cryptic species have not been identified in the UK, despite a study that examined 79 individuals of the subgenus *Avaritia* using a 472 bp region of the mitochondrial cytochrome *c* oxidase subunit 1 gene (*cox*1) across ten geographically disparate sampling points [25].

All four established members of *Culicoides* (*Avaritia*) in northwestern Europe have been implicated in transmission of SBV. Schmallenberg virus RNA has been detected in homogenates of heads removed from field-collected *C. obsoletus*, *C. scoticus* and *C. chiopterus* individuals in the Netherlands [15], and additionally within heads of *C. dewulfi* collected in Belgium [17]. A key research limitation, however, is that techniques have not been developed to artificially blood-feed field-collected species of Palaearctic *Culicoides* through membranes under conditions of biological containment [30, 31]. This not only limits studies attempting to standardise population susceptibility to infection across the region, but also prevents the establishment of colony and cell lines of these species as a resource [32].

In this study we examined methods of blood-feeding species of field-collected *Culicoides* in the UK to provide a standard for comparative studies of vector competence across Europe. We then examined vector competence in a single population of *Culicoides* collected at a stable in Surrey, UK. To our knowledge, for the first time, we link standard detection of disseminated infections with haplotype-based identification based on Sanger-sequencing of the DNA barcode region of the mitochondrial cytochrome *c* oxidase subunit 1 (*cox*1) gene. This not only provides preliminary data concerning susceptibility rates to SBV infection in known vector species of *Culicoides* in northwestern Europe, but also provides additional data concerning the complement of species and *cox*1 haplotypes that are present in the UK, with consequences for epidemiological studies of arbovirus transmission and epidemiology.

## Methods

### Study site and collections

Specimens of *Culicoides* were collected at a site in close proximity to horses at a stable in Surrey (51°28.97N; -06°52.03W) during 2012 and 2013 using two 8 W ultraviolet Onderstepoort Veterinary Institute (OVI) light-suction traps (Agricultural Research Council - Onderstepoort Veterinary Institute, Pretoria, South Africa) [33]. Collections were made overnight in a 500 ml plastic beaker partially filled with damp paper hand-towel in an attempt to minimise the effects of desiccation on collected *Culicoides*. Following collection, collected insects were allowed to emerge from the beaker against a glass plane in front of natural daylight allowing selected aspiration of free-flying *Culicoides* for use during later experiments. These were transferred to netted, cylindrical, card 64 mm diameter pillboxes (Watkins and Doncaster, Stainton, UK) for use in later experiments.

### Effect of membrane type on blood-feeding rates

All blood-feeding studies were conducted in 2012 using an artificial feeding system (Hemotek), with each feeder unit calibrated to warm the blood-meal to 37 °C. Prior to being offered a blood-meal, all *Culicoides* were incubated for 96 h following collection, at 25 ± 1 °C and ≥ 50% relative humidity (RH) with *ad libitum* access to 10% (*w/v*) sucrose solution (Sigma-Aldrich, Gillingham, UK) provided *via* a saturated cotton wool pledglet placed on the netted tops of pillboxes. Pledglets were removed 24 h prior to offering *Culicoides* a blood meal. Three membrane types were compared, used in conjunction with an artificial feeding system (Hemotek). Blood-meal reservoirs containing approximately 3 ml of defibrinated equine blood (TCS Biosciences, Botolph Claydon, UK) were covered with either a (i) stretched skins taken from one-day old-chicks; (ii) collagen membrane (Hemotek); or (iii) stretched Parafilm M® membrane (Bemis Company Inc., Neenah, WI, USA). Each membrane type was assessed in duplicate for each round of blood-feeding with membrane types randomly allocated two of the six feeders units per blood-feeding round. A total of nine days of duplicate replicates were completed for each membrane type. Blood meals were offered for a total of 45 min, after which the contents of the pillboxes were killed *via* prolonged exposure to cold and *Culicoides* were identified to species or subgenus level according to their morphology with reference of appropriate keys [24]. In addition specimens were classified according to abdominal pigmentation (unpigmented or pigmented); blood-fed or gravid in the case of females [34, 35], or as males. Blood-feeding success rates were calculated using only unpigmented, pigmented and blood-fed females. The effect of membrane type and date of blood-feeding on the number of blood-fed *Culicoides* was assessed using a Kruskal-Wallis test. Where the Kruskal-Wallis test was significant (*P* < 0.05), differences between factor levels were explored using pairwise Wilcoxon rank-sum tests.

### Vector competence

All vector competence studies were conducted in August-October 2013. The SBV strain used for studies of infection was provided by IZS Teramo from an isolation originally made by the Friedrich-Loeffler-Institute, Isle of Riems, Germany [1]. This SBV strain had been passaged once through a *C. sonorensis* derived cell line and four times through a baby hamster kidney (BHK-21) cell line and then adjusted to a titre of 10^6^ tissue culture infectious dose 50 (TCID_50_) using defibrinated equine blood (TCS Bioscience). All blood-feeding and sorting of *Culicoides* were carried out in a biosecure glove-box. Blood-virus mixes were offered using either (i) an artificial feeding system (Hemotek) as described above with a stretched Parafilm M® membrane (Bemis Company Inc.), or *via* (ii) a saturated cotton wool pledglet placed directly on top of the net of the pillboxes [31].

Blood meals were offered for a total of 45 min, after which blood-fed *Culicoides* were selected under light CO_2_ anaesthesia and then incubated in netted, cylindrical, card 64 mm diameter pillboxes (Watkins and Doncaster) for eight days at 25 °C with *ad libitum* access to 10% (*w/v*) sucrose solution (Sigma-Aldrich) provided *via* a cotton wool pledglet placed on top of the netted pillboxes. *Culicoides* surviving the incubation period were then selected under CO_2_ anaesthesia and transferred to 1.5 ml Eppendorf tubes containing 70% ethanol and stored at 4 °C prior to further analysis.

Specimens were individually removed from storage in 70% ethanol and decapitated using a sterile needle (Monoject™ hypodermic needle, 18g × 1.5; Covidien, Minneapolis, MN, USA). Heads were then transferred individually to 1.5 ml microcentrifuge tubes containing 100 μl Schneider’s Drosophila Media (Gibco™, Paisley, UK) and homogenised using disposable polypropylene pestles (Sigma-Aldrich). The remaining abdomen and thorax of each individual was transferred to a collection microtube (Qiagen, Manchester, UK) containing 100 μl of Schneider’s Drosophila Media and a 3 mm stainless steel bead (Dejay Distribution Ltd., Launceston, UK) and homogenised for 1 min at 25 hz using a TissueLyser® (Qiagen).

### Schmallenberg virus detection

Total nucleic acid was extracted from the homogenates of *Culicoides* heads using a Universal Biorobot (Qiagen) with a QIAamp® All Nucleic Acid MDx Kit (Qiagen) following the manufacturer’s recommended instructions. Schmallenberg virus RNA in the resultant extractions was the assessed using a semi-quantitative RT-qPCR targeting the S segment of the genome [1, 20]. A C_q_ cut-off value of < 35 was used to define SBV infection [15].

### *cox*1 DNA barcode assay

Amplification of a 658 bp fragment of the DNA barcoding region of the mitochondrial* cox*1 gene [36] was achieved by polymerase chain reaction (PCR). Reactions were performed in a total volume of 25 μl consisting of 13.65 μl nuclease-free water (Qiagen), 2.5 μl 10× PCR Buffer (Life Technologies, Paisley, UK), 0.75 μl 50 mM MgCl_2_ (Life Technologies)_,_ 0.5 μl 10 mM dNTP mix (Life Technologies); 0.1 μl Platinum® *Taq* DNA polymerase (Life Technologies), 1.25 μl of the 20 μM forward primer, 1.25 μl of the 20 μM reverse primer and 5.0 μl of template DNA (approximately 5–25 ng DNA) per reaction. Amplification of the DNA barcode region was initially attempted using the following primer pair: LCO1490 (5′-GTC AAC AAA TCA TAA AGA TAT TGG-3′ [37]) and HCO2198 (5′-TAA ACT TCA GGG TGA CCA AAA AAT CA-3′ [37]). If the DNA barcode region could not be sucessfully amplified using primers LCO1490 and HCO2198, the above assay was repeated using the following alternative primer pair: LepF1 (5′-ATT CAA CCA ATC ATA AAG ATA TTG G-3′ [38]) and LepR1 (5′-TAA ACT TCT GGA TGT CCA AAA AAT CA-3′ [38]). Positive and negative controls for the amplification reactions were carried out at every PCR round. The PCR cycling conditions for all DNA Barcode assays were as follows: an initial denaturation step at 94 °C for 2 min followed by 35 cycles of 94 °C for 30 s, 46 °C for 30 s, 72 °C for 1 min, and a final extension step at 72 °C for 10 min. PCR products were visualised using ultraviolet light and 2% agarose gels containing SYBR® Safe DNA Gel Stain (Invitrogen, Paisley, UK). Successful amplification of the* cox*1 DNA barcode region was indicated by the presence of a band at approximately 720 bp for both primer pair LCO1490 and HCO2198 and primer pair LepF1 and LepR1, identified by comparison with E-Gel® Low Range Quantitative DNA Ladder (100–2000 bp; Life Technologies).

### PCR purification and* cox*1 sequencing

Dimer formation from the primers was not observed and purification of the remaining PCR product was performed using a GFX^TM^ PCR DNA and Gel Band Purification Kit (GE Healthcare Life Sciences, Little Chalfont, UK), following the manufacturer’s recommended guidelines. The amplicons were sequenced bi-directionally using BigDye Terminator v3.1 Cycle Sequencing kit (Applied Biosystems, Paisley, UK) in a 3730 DNA Analyzer (Applied Biosystems) according to the manufacturer’s instructions using the primer pairs as per the PCR amplification either HCO2198 and LCO1490, or LepF1 and LepR1.

### Phylogenetic analysis

Electropherograms were edited and forward and reverse sequences assembled and trimmed to remove primer sequence using CodonCode Aligner v. 5.1.5 (CodonCode Aligner, Centerville, MA, USA). Corresponding specimen collection data and DNA sequences including electropherograms have been made publically available *via* the Barcode of Life Data System (BOLD) [39] as dataset DS-CUSBV (dx.doi.org/10.5883/DS-CUSBV), DNA sequences were also submitted to the GenBank database (accession numbers KT186808–KT186881).

Consensus sequences were compared to previously published sequences in GenBank using the standard nucleotide BLAST tool [40], in addition to comparison to as yet unreleased sequence data in the BOLD database [39] using the Barcode Identification Engine in BOLD v3. Obsoletus group *cox*1 sequences which overlapped the DNA barcode region [36] by at least 390 bp were obtained from both BOLD (*n* = 25) and GenBank (*n* = 393) and included in the phylogenetic analysis (Additional file 1: Table S1), these sequences were selected in order to assess if the haplotypes identified within this study were concordant with those identified from other geographical regions.

All sequences were aligned using MUSCLE [41] and the alignment quality checked using GUIDANCE [42] (100 bootstraps). All included sequences were aligned with a high degree of confidence (GUIDANCE alignment score > 0.999). jModelTest [43, 44] v. 2.1.7 was then used to determine the most suitable DNA substitution model among the 24 models that can be implemented in MrBayes [45, 46]. The model with the lowest Bayesian information criterion (BIC) and Akaike information criterion (AIC), the Hasegawa-Kishino-Yano with gamma-distribution rates (HKY+G) [47], was considered to best describe the nucleotide substitution pattern.

The phylogenetic relationships among taxa were resolved using a Bayesian inference (BI) approach using the HKY+G nucleotide substitution model rooted on the partial *cox*1 sequence of *C. imicola* Kieffer (GenBank: KT307824) [48]. The BI tree was constructed using MrBayes v. 3.2.2 [46, 49] and twenty million tree generations in four chains were run, sampling every 1000th and discarding the first 25%, before constructing a 50% majority rule consensus tree reporting Bayesian posterior probabilities. Convergence was assessed using AWTY [50]. The BI trees were visualised using the packages Ape v. 3.2 [51] and Phytools v. 0.4–60 [52] implemented in R v. 3.1.2 [53].

Relationships between the observed haplotypes within the *C. obsoletus* specimens for which SBV vector competence data were available (*n* = 67) (GenBank: KT186812–KT186878), were assessed by constructing Median-Joining networks. Roehl haplotype data files (RDF) were created with DnaSP v5.10 [54] and imported into Network v4.6.1.2 [55]. Networks were calculated with the Median-Joining algorithm [56] with equal weights for all characters, using maximum parsimony [57] post-processing. Intra- and inter-specific uncorrected percent nucleotide sequence distances, were generated using the packages Spider v. 1.3–0 [58] and Ape v. 3.2 [51] implemented in R v. 3.1.2 [53]. Missing nucleotides were treated in all sequence comparisons using a pairwise deletion option.

## Results

### Effect of membrane type on blood-feeding

A total of 6029 *Culicoides* were used in the experiments to investigate the effect of membrane type on *Culicoides* blood-feeding rates: 4681 specimens of the Obsoletus complex including the morphologically cryptic *C. obsoletus* (Meigen) and *C. scoticus* Downes & Kettle; 157 *C. dewulfi*; 4 *C. chiopterus*; 127 *C. pulicaris* (L.); 138 *C. punctatus* (Meigen), 4 *C. impunctatus* Goetghebuer; 321 *C. achrayi* Kettle & Lawson; 537 *C. festivipennis* Kieffer; 60 individuals from other *Culicoides* species (Additional file 2: Table S2). Excluding gravid females and males, a total of 4270 specimens of the Obsoletus complex; 121 *C. dewulfi*; 2 *C. chiopterus*; 96 *C. pulicaris*; 85 *C. punctatus*; 4 *C. impunctatus*; 255 *C. achrayi*; 43 *C. festivipennis* were exposed to the membrane systems during trials (Table 1). Only blood-feeding rates of the species of the Obsoletus complex provided sufficient sample sizes for further statistical analysis. Membrane type had a significant (Kruskal-Wallis test: *χ*^2 ^= 27.1, *df* = 2, *P* < 0.0001) effect on Obsoletus complex *Culicoides* blood-feeding rate, with both chick skin (Wilcoxon rank-sum test: *W* = 27, *P *< 0.0001) and Parafilm M® (Wilcoxon rank-sum test: *W* = 297, *P* < 0.0001) membrane resulting in a significantly higher blood-feeding rate that the collagen membrane. There was no significant (Wilcoxon rank-sum test: *W* = 180, *P *= 0.57) difference in blood-feeding rate between Obsoletus complex *Culicoides* fed *via* chick skin and Parafilm M® membrane. The day of blood-feeding also had no significant effect (Kruskal-Wallis test: *χ*^2 ^= 2.7, *df* = 7, *P *= 0.91) on blood-feeding rates.

**Table 1:** Comparison of membrane type with an artificial feeding system (Hemotek, UK) on *Culicoides* blood-feeding rate (blood-feeding rate with range and total number offered a blood meal shown in parentheses). Data exclude gravid females and males exposed to blood-feeding system

*Culicoides* spp./ Species complex	Blood-feeding rate (%)		
	Chick skin	Collagen	Parafilm M®
Obsoletus complex (*n* = 4270)	8.0 (0–16.0; *n* = 1384)	0 (*n* = 1331)	5.0 (0–32.0; *n* = 1555)
*C. dewulfi* Goetghebuer (*n* = 121)	7.0 (0–33.0, *n* = 27)	0 (*n* = 50)	0 (*n* = 44)
*C. chiopterus* (Meigen) (*n* = 2)	–	–	0 (*n* = 1)
*C. pulicaris* (L.) (*n* = 34)	0.03 (0–33.0; *n* = 34)	–	–
*C. punctatus* (Meigen) (*n* = 85)	0 (*n* = 37)	0 (*n* = 19)	0.10 (0–100.0; *n* = 29)
*C. impunctatus* Goetghebuer (*n* = 4)	0 (*n* = 1)	0 (*n* = 2)	0 (*n* = 1)
*C. achrayi* (Kettle & Lawson) (*n* = 255)	8.0 (0–33.0; *n* = 85)	0 (*n* = 87)	2.0 (0–25.0; *n* = 83)
*C. festivipennis* Kieffer (*n* = 43)	30.8 (0–100.0; *n* = 13)	0 (*n* = 6)	0 (*n* = 24)
Other *Culicoides* spp. (*n* = 4)	0 (*n* = 4)	–	–
Total blood-fed (%)	119 (7.9)	0 (0)	76 (4.3)

### Vector competence

In total, 147 *Culicoides* survived the eight day incubation period post-feeding on the blood-virus mix through an artificial membrane; of these 13 (11.9%) of *Culicoides* (*Avaritia*) contained significant quantities of SBV RNA within their heads (C_q_ ≤ 40) (Table 2). Using a more conservative cut-off level for detection of SBV RNA (C_q_ ≤ 35), this number fell to 8 (7.3%). In total, 104 *Culicoides* were successfully fed using pledglet blood-feeding, of which 94 were from the subgenus *Avaritia* (Table 2). Of these, 2 (2.1%) contained significant quantities of SBV RNA in their heads (C_q_ ≤ 40) (Tables 2, 3). Using a more conservative cut-off level for detection of SBV RNA (C_q_ ≤ 35), this number fell to 1 (1.1%). In *C. pulicaris* fed using a membrane method both processed individuals tested positive for disseminated SBV RNA of which one had a C_q_ value lower than the conservative cut-off (Table 3). Heads from no other species examined contained detectable levels of SBV RNA (Table 2).

**Table 2:** Susceptibility to infection with Schmallenberg virus for *Culicoides* (*Avaritia*) collected at a single site in the UK

Subgenus *Avaritia*	Feeding method	
	Membrane^a^	Pledglet^a^
	109 (13)	94 (2)
*C. pulicaris* (L.)	2 (2)	4 (0)
*C. punctatus* (Meigen)	4 (0)	6 (0)
*C. achrayi* (Kettle & Lawson)	23 (0)	–
*C. festivipennis* Kieffer	9 (0)	–
Total	147 (15)	104 (2)

^**a**^*n* with total with a quantification cycle (C_q_) ≤ 40 in parenthesis

**Table 3:** Observed quantification cycle (C_q_) values for Schmallenberg virus (SBV) in field-collected *Culicoides*. Includes associated *Culicoides* species characterisation and blood-feeding method (– indicates no sequence data available; Obsoletus complex includes *C. obsoletus* (Meigen) and *C. scoticus* Downes & Kettle)

Species	BIN^a^	Blood-feeding method	SBV C_q_ value	*Culicoides* DNA barcode		
				Sample ID	GenBank ID	BOLD process ID
*C. obsoletus* (Meigen)	BOLD:AAO7718	Membrane	32.95	TPI:ENT:#0000313	KT186817	CUSBV066–15
		Membrane	32.84	TPI:ENT:#0000314	KT186816	CUSBV067–15
		Membrane	33.64	TPI:ENT:#0000315	KT186815	CUSBV068–15
		Membrane	31.00	TPI:ENT:#0000316	KT186814	CUSBV069–15
		Membrane	37.88	TPI:ENT:#0000319	KT186813	CUSBV072–15
		Membrane	37.44	TPI:ENT:#0000320	KT186812	CUSBV073–15
		Membrane	36.11	TPI:ENT:#0000321	KT186860	CUSBV074–15
		Membrane	38.10	TPI:ENT:#0000322	KT186859	CUSBV075–15
		Membrane	32.61	TPI:ENT:#0000323	KT186858	CUSBV076–15
	BOLD:AAM6198	Membrane	31.92	TPI:ENT:#0000325	KT186863	CUSBV078–15
*C. scoticus* Downes & Kettle	BOLD:AAZ3985	Pledglet	37.88	TPI:ENT:#0000317	KT186880	CUSBV070–15
		Pledglet	33.83	TPI:ENT:#0000318	KT186881	CUSBV071–15
Obsoletus complex^b^	–	Membrane	34.00	–	–	–
	–	Membrane	27.12	–	–	–
	–	Membrane	37.67	–	–	–
*C. pulicaris* (L.)^b^	–	Membrane	28.17	–	–	–
	–	Membrane	35.95	–	–	–

^a^Barcode Index Numbers (BINs) [49] assigned within the Barcode of Life Database (BOLD) [39] for specimens collected within this study: specimens TPI:ENT:#0000248–TPI:ENT:#0000262; TPI:ENT:#0000264–TPI:ENT:#0000283; TPI:ENT:#0000285–TPI:ENT:#0000286; TPI:ENT:#0000288–TPI:ENT:#0000312 [GenBank: KT186808–KT186811; KT186818–KT186857; KT186861–KT186862; KT186864–KT186879]

^b^Species identification based on morphology only

### Phylogenetic analysis

**Fig. 1:**
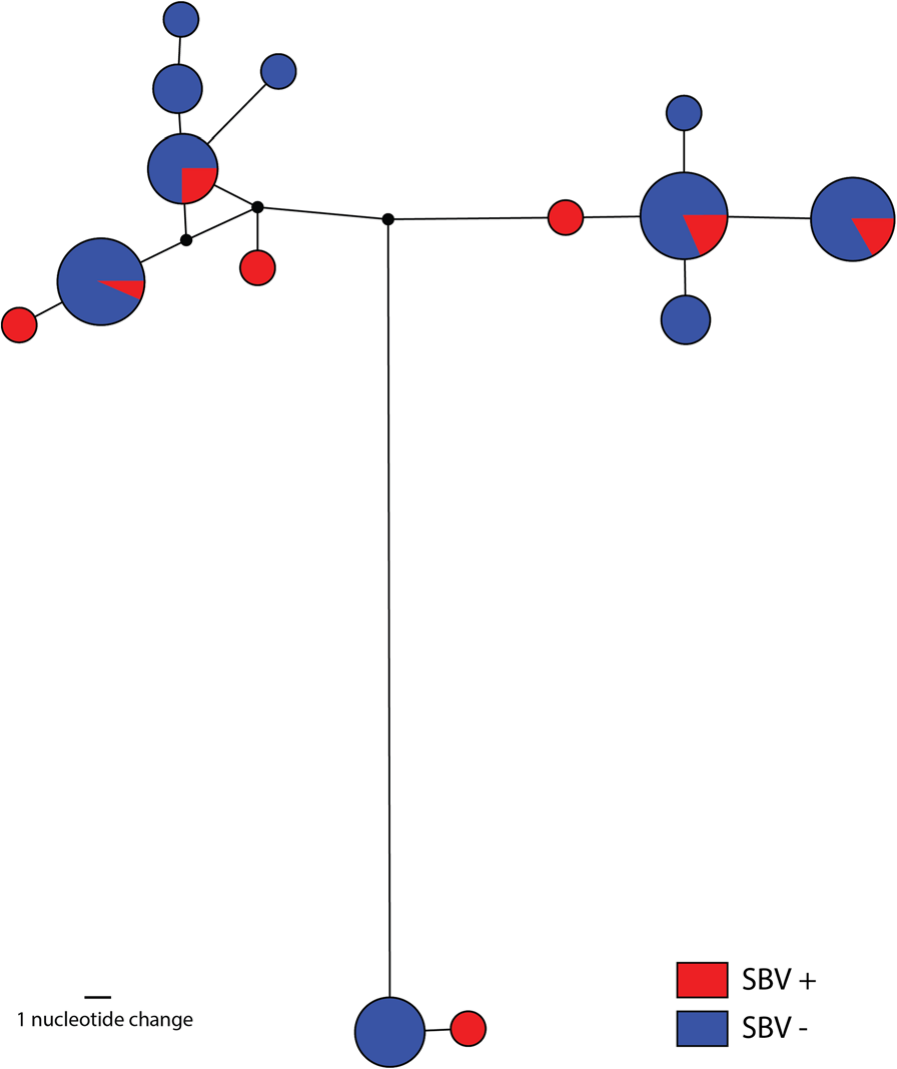
Most parsimonious median-joining network (ε = 0) depicting phylogenetic relationships among *C. obsoletus cox*1 haplotypes. The size of each circle is proportional to the corresponding haplotype frequency. Circles are coloured to represent the proportion of specimens which showed a positive (red) and negative (blue) SBV response as determined by qPCR. Branch lengths are proportional to the number of nucleotide changes between haplotypes. Black circles indicate median vectors (*mv*) that represent hypothetical missing or unsampled ancestral haplotypes

Full length primer truncated (658 bp) DNA barcode sequences (*n* = 74) were obtained from three species of the subgenus *Avaritia*: *C. obsoletus* (*n* = 67), *C. scoticus* (*n* = 3) and *C. dewulfi* (*n* = 4). No insertions, deletions, amino acid frame shifts or stop codons were observed among these sequences, and their translations, indicating that pseudogenes were not present within the alignments. DNA barcodes were obtained from 12 of a total of 15 specimens containing SBV RNA in their heads (C_q_ ≤ 40): ten from membrane-fed individuals and two from pledglet-fed individuals (Table 3). Ten were identified as *C. obsoletus* (Table 3), there was, however, no apparent association between haplotype and SBV vector competence within this species (Fig. 1). DNA from a further three specimens could not be amplified successfully and these were identified on the basis of morphology only as belonging to the Obsoletus complex (Table 3). DNA barcodes for an additional 46 membrane-fed *Culicoides* and 17 pledglet-fed *Culicoides* lacking detectable SBV RNA (C_q_ ≥ 40) were also generated and analysed (Table 3).

**Fig. 2:**
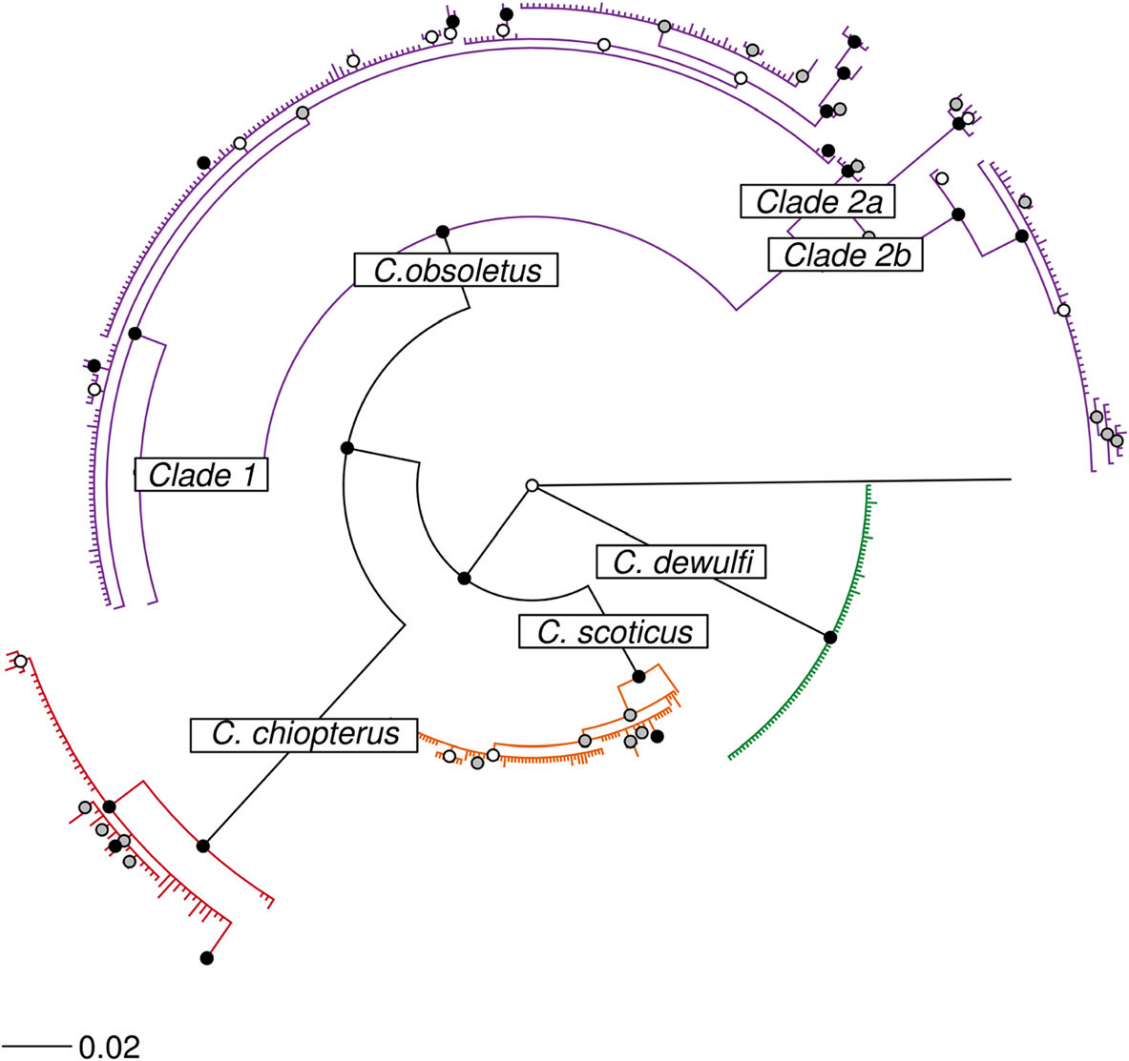
Simplified Bayesian inference phylogenetic tree inferred from* cox*1 DNA barcode sequences with species assignments indicated. Bayesian posterior probability node support values ≥ 0.95 are shown as solid black circles at nodes, 0.75–0.94 shown as black outline circles with grey fill at nodes and < 0.75 shown as black outline circles with white fill at nodes

**Fig. 3:**
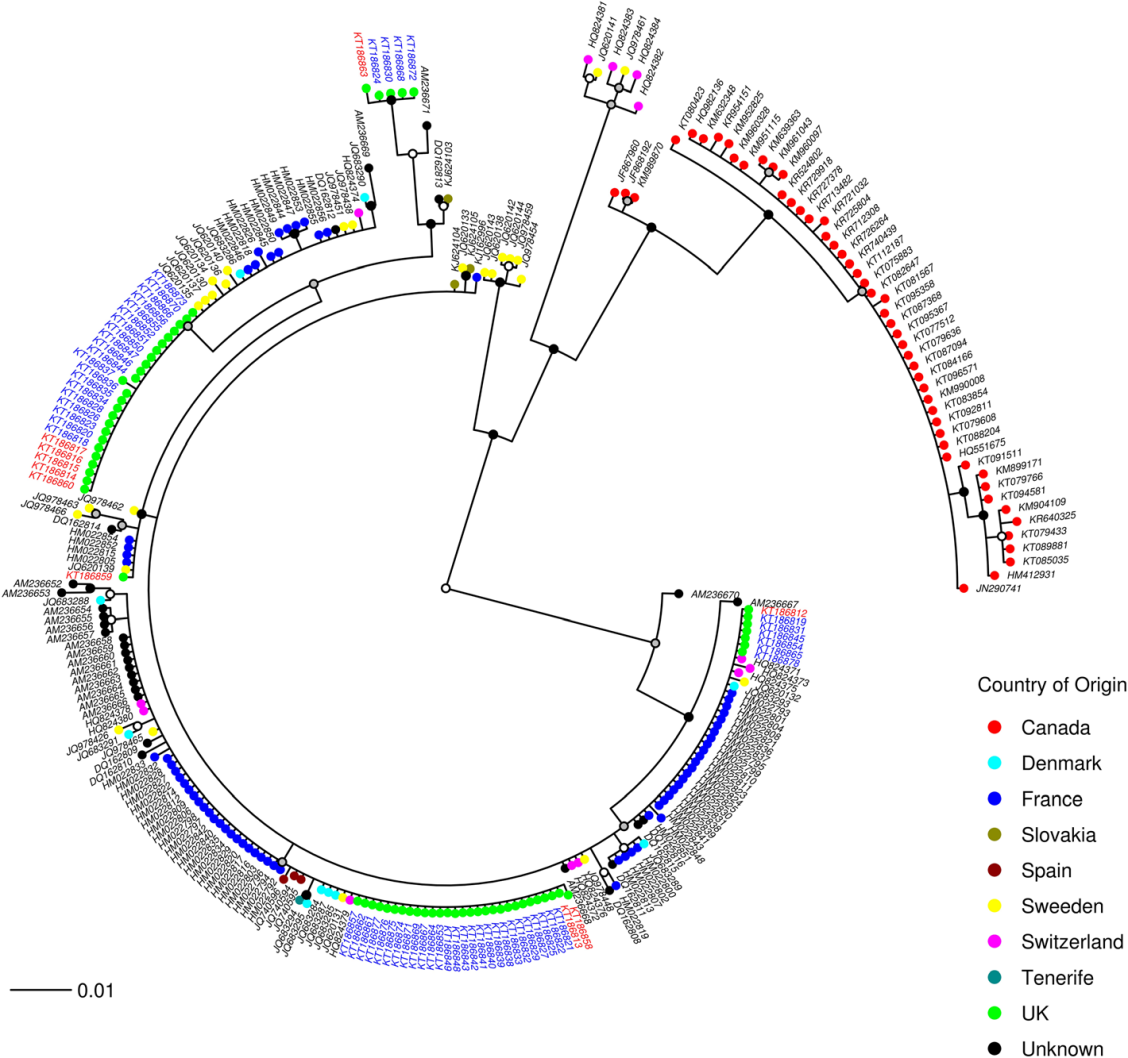
Bayesian inference phylogenetic tree inferred from *C. obsoletus cox*1 DNA barcode sequences. Bayesian posterior probability node support values ≥ 0.95 are shown as solid black circles at nodes, 0.75–0.94 shown as black outline circles with grey fill at nodes and < 0.75 shown as black outline circles with white fill at nodes. Geographical origin of specimens indicated by a coloured circle preceding the GenBank accession number for the sequence. Schmallenberg virus (SBV) vector competence indicates by colour of the GenBank accession number tip labels (red, positive; blue, negative; black, unknown). Clades shown represented in purple in Fig. 2

**Fig. 4:**
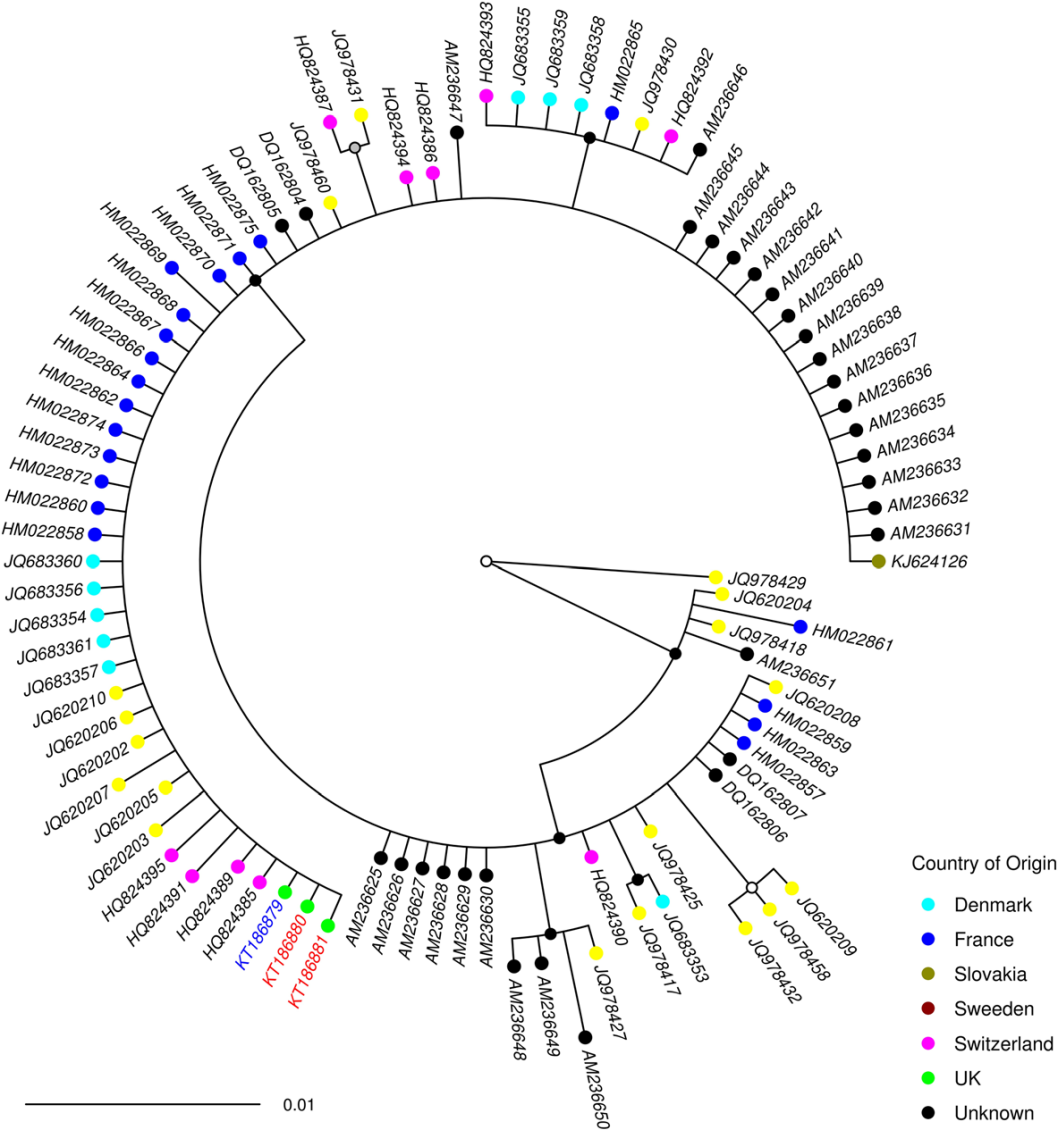
Bayesian inference phylogenetic tree inferred from *C. scoticus cox*1 DNA barcode sequences. Bayesian posterior probability node support values ≥ 0.95 are shown as solid black circles at nodes, 0.75–0.94 shown as black outline circles with grey fill at nodes and < 0.75 shown as black outline circles with white fill at nodes. Geographical origin of specimens indicated by a coloured circle preceding the GenBank accession number for the sequence. Schmallenberg virus (SBV) vector competence indicates by colour of the GenBank accession number tip labels (red, positive; blue, negative; black, unknown). Clades shown represented in orange in Fig. 2

**Fig. 5:**
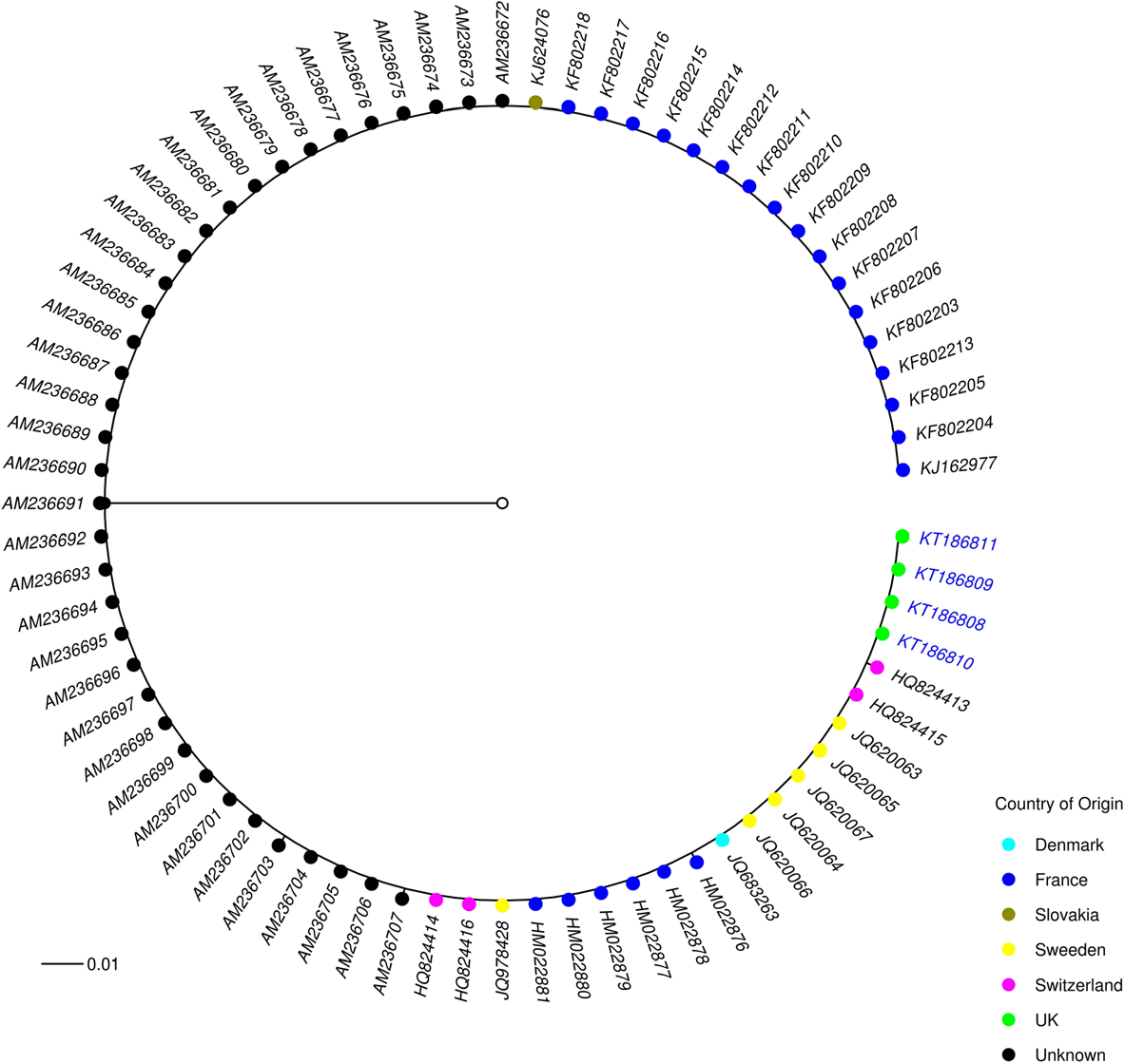
Bayesian inference phylogenetic tree inferred from *C. dewulfi cox*1 DNA barcode sequences. Bayesian posterior probability node support values ≥ 0.95 are shown as solid black circles at nodes, 0.75–0.94 shown as black outline circles with grey fill at nodes and < 0.75 shown as black outline circles with white fill at nodes. Geographical origin of specimens indicated by a coloured circle preceding the GenBank accession number for the sequence. Schmallenberg virus (SBV) vector competence indicates by colour of the GenBank accession number tip labels (red, positive; blue, negative; black, unknown). Clade shown represented in green in Fig. 2

**Fig. 6:**
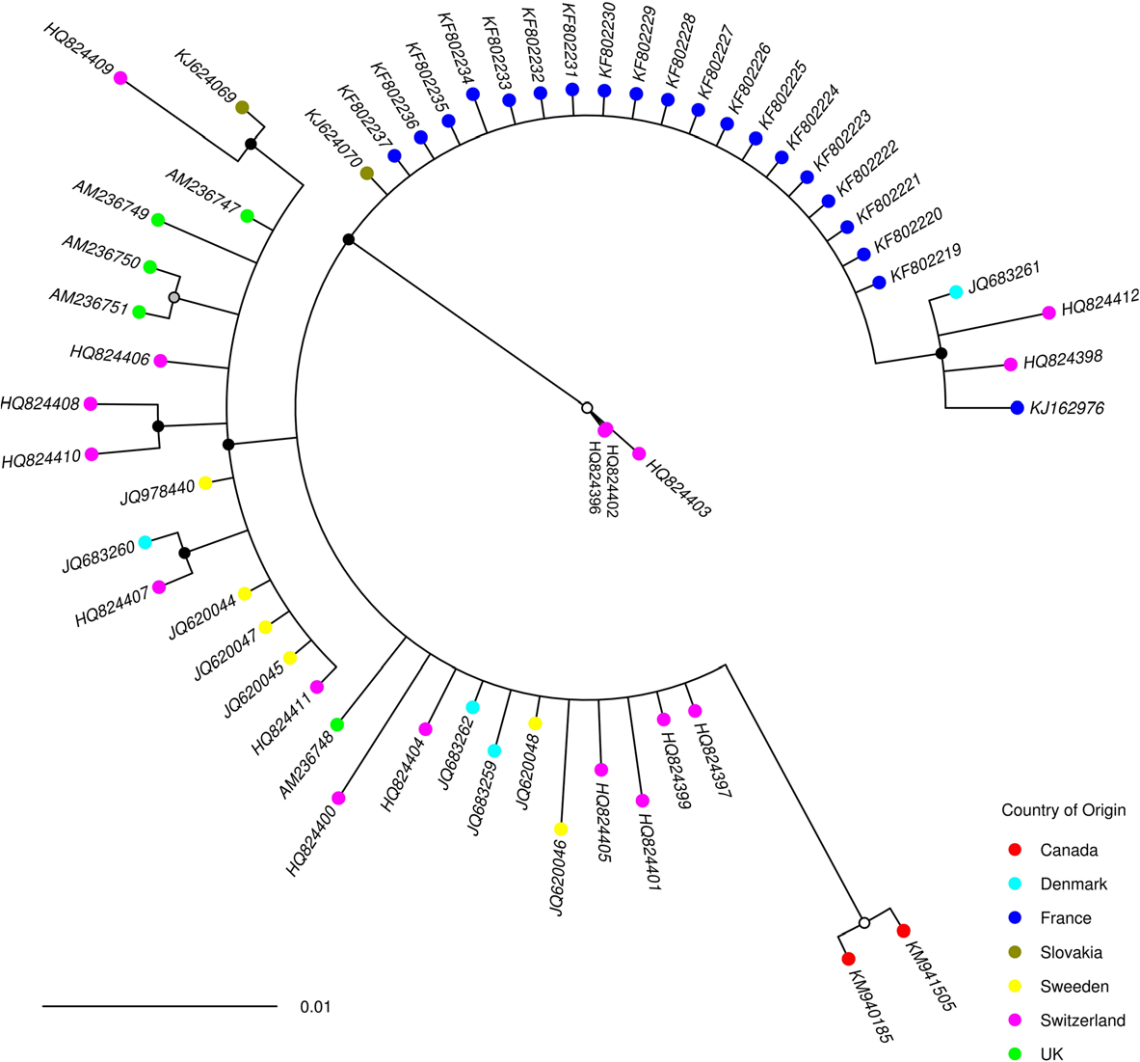
Bayesian inference phylogenetic tree inferred from *C. chiopterus cox*1 DNA barcode sequences. Bayesian posterior probability node support values ≥ 0.95 are shown as solid black circles at nodes, 0.75–0.94 shown as black outline circles with grey fill at nodes and < 0.75 shown as black outline circles with white fill at nodes. Geographical origin of specimens indicated by a coloured circle preceding the GenBank accession number for the sequence. Schmallenberg virus (SBV) vector competence indicates by colour of the GenBank accession number tip labels (red, positive; blue, negative; black, unknown). Clades shown represented in red in Fig. 2

Phylogenetic analysis of the DNA barcodes from specimens generated within this study together with *cox*1 sequences obtained from GenBank and BOLD (Additional file 1: Table S1) indicated that the species clades represented in the BI phylogeny were concordant with morphological identifications generated using the key of Campbell & Pelham-Clinton [24] (Figs. 2, 3, 4, 5, 6). The *cox*1 sequences obtained from GenBank and BOLD overlapped the 658 bp DNA barcodes generated in this study by between 391 bp and 658 bp.

**Fig. 7:**
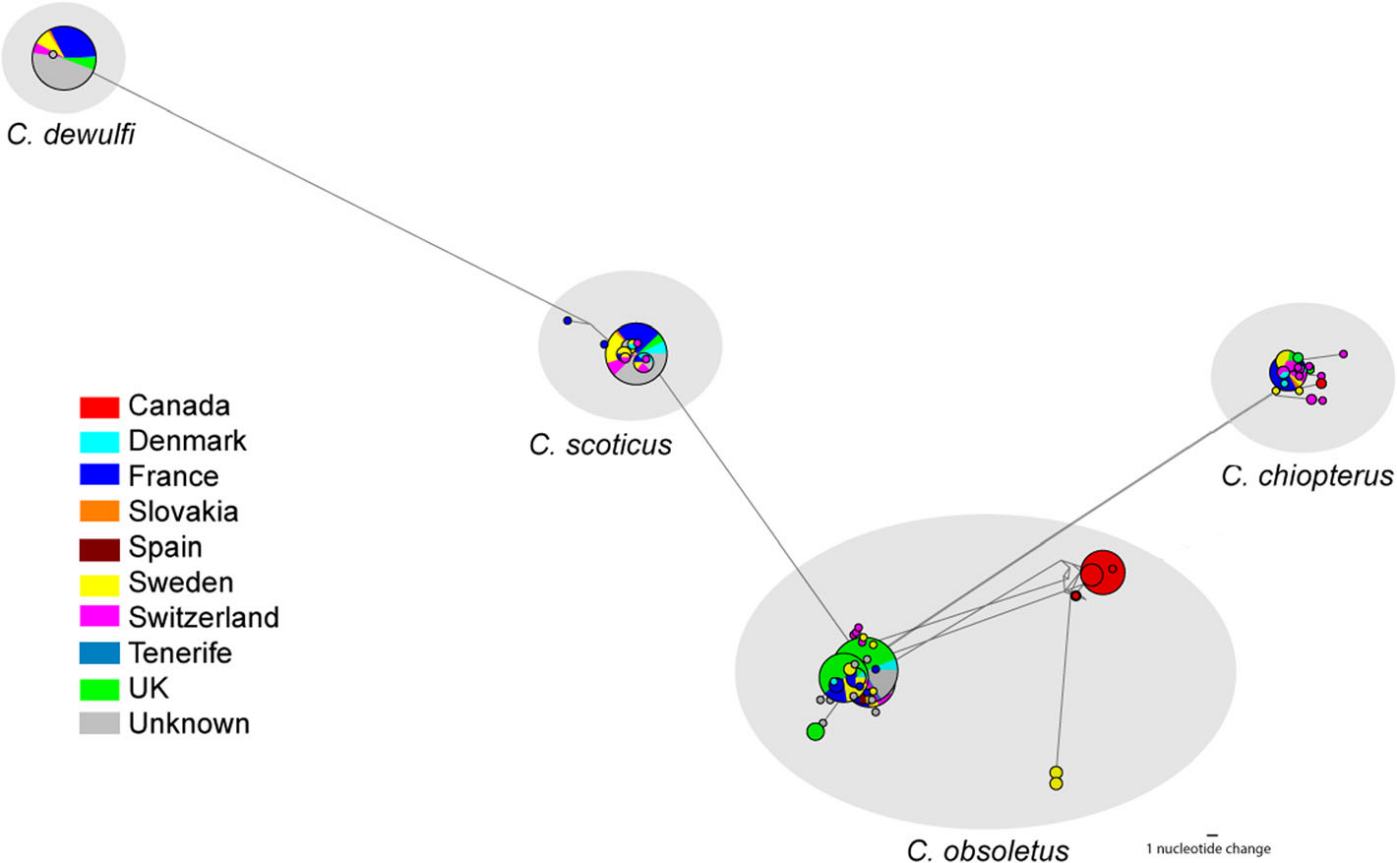
Most parsimonious median-joining network (ε = 0) depicting phylogenetic relationships *cox*1 haplotypes. The size of each circle is proportional to the corresponding haplotype frequency. Branch lengths are proportional to the number of nucleotide changes between haplotypes

Deep interspecific differences within the *cox*1 DNA barcode region were present between the majority of *Culicoides* species present within the phylogenetic analysis (Table 4, Figs. 2, 7; Additional file 1: Figures S1, S2). The greatest intraspecific nucleotide sequence difference was between *C. dewulfi* and *C. chiopterus* [mean 22.5% (range: 20.5–23.5%)], while the least was between *C. obsoletus* and *C. scoticus* [mean 12.2% (range: 10.9–14.3%)] (Table 4; Additional file 1: Figure S2). Sequence differences symptomatic of cryptic species diversity were, however, present within *C. obsoletus* (Table 4; Additional file 1: Figure S2). Intraspecific nucleotide sequence differences were observed between specimens nominally identified as *C. obsoletus* ranging from 0 to 12.3%.

**Table 4:** Nucleotide sequence distances between UK *Culicoides cox*1 DNA barcodes. Uncorrected percent nucleotide sequence distances, mean with range shown in parentheses split by species including the proposed clades of *C. obsoletus* (see Fig. 2). Intraspecific distances are shown in bold along the diagonal, interspecific distances are shown below the diagonal. The number of specimens per species (*n*) is shown in brackets followed by the number of specimens originating from this study; and the number originating from GenBank in parentheses

	*C. chiopterus*	*C. dewulfi*	*C. obsoletus*	*C. obsoletus* Clade 1	*C. obsoletus* Clade 2a	*C. obsoletus* Clade 2b	*C. scoticus*
*C. chiopterus* [56 (0; 56)]	**0.8 (0.0–3.3)**						
*C. dewulfi* [75 (4; 71)]	22.5 (20.5–23.5)	**0.1 (0.0–0.9)**					
*C. obsoletus* [169 (124; 145)]	14.9 (13.2–18.3)	19.3 (17.5–22.8)	**4.1 (0.0–12.3)**				
*C. obsoletus* Clade 1 [206 (67; 139)]	14.6 (13.2–16.4)	18.8 (17.5–20.6)		**0.9 (0.0–4.4)**			
*C. obsoletus* Clade 2a [6 (0; 6)]	16.2 (14.1–18.3)	21.0 (19.4–22.8)		9.4 (7.6–11.7)	**1.7 (0.0–8.1)**		
*C. obsoletus* Clade 2b [57 (57; 0)]	14.7 (13.8–15.5)	20.2 (19.6–21.0)		11.1 (10.2–12.3)	8.6 (7.9–9.3)	**0.6 (0.2–0.9)**	
*C. scoticus* [92 (3; 89)]	14.0 (12.0–15.8)	18.2 (16.9**–**19.3)	12.2 (10.9–14.3)	12.1 (10.9**–**13.7)	11.6 (14.3)	12.1 (11.6–12.9)	**0.4 (0.0–3.3)**

Barcode Index Number (BINs) [49] assigned within BOLD [39] for *C. scoticus* (BOLD:AAZ3985, *n* = 3) and *C. dewulfi* (BOLD:AAZ3991, *n* = 4) were concordant across all specimens sequenced within this study, however discordant BINs were observed for the *C. obsoletus* specimens sequenced within this study (Concordant BINs exhibit > 2% nucleotide sequence divergence [49]). This resulted in two BINs (BOLD:AAM6198, *n =* 5*;* BOLD:AA07718, *n* = 62) being assigned for specimens putatively identified as *C. obsoletus* thereby indicating the potential presence of two distinct taxa (see Table 3, dataset DS-CUSBV: dx.doi.org/10.5883/DS-CUSBV).

## Discussion

This study has developed artificial blood-feeding techniques for *Culicoides* in the UK and then demonstrated susceptibility to infection with SBV in multiple haplotypes of *C. obsoletus* and *C. scoticus* under laboratory conditions. The results confirm those that identified SBV RNA in the heads of these species in close proximity to ruminants in the Netherlands [15], Belgium [17] and France [14]. Blood-feeding of *Culicoides* on pledglets was also demonstrated to underestimate rates of susceptibility to infection, as found previously with BTV [30, 31]. The sequencing of the DNA barcode region (658 bp) of the *cox*1 gene for 67 individuals putatively identified as *C. obsoletus* from a single site in the UK provides potential evidence of cryptic diversity, however, how this relates to the morphologically cryptic species previously described in the Netherlands, Sweden and Switzerland [27–29, 59] remains unclear.

Blood-feeding techniques developed within this study enabled the first membrane-based artificial feeding experiments using northwestern European *Culicoides* since the 1980s [60]. Unlike the original method, which relied upon the use of populations in close proximity to contained facilities, the technique had the advantage of including an incubation step that precluded the inclusion of naturally blood-fed individuals from traps and can be used with light-suction trap collections from a wide geographical area. While blood-feeding rates remained poor, this development is useful in creating a platform for both vector competence experimentation and wider vector-virus interaction studies in addition to laboratory-based studies of bionomics for which little data is available for the subgenus *Avaritia* in northwestern Europe [32]. Further investigation of techniques to improve feeding rates is now required as provided for *C. impunctatus* in northwestern Europe [61, 62].

A high rate of vector competence for SBV in *Culicoides* is hypothesised to be at least partly responsible for driving the rapid geographic spread of the virus in Europe [14, 15, 63]. While the preliminary data provided in the current study shows that susceptibility to infection with SBV is low, significant variation in vector susceptibility to infection according to population tested has been documented for *Culicoides* of the subgenus *Avaritia* in the UK for BTV [30]. Hence, further screening of *Culicoides* populations utilising multiple populations across Europe would be beneficial in improving our understanding of transmission and in informing mathematical modelling studies [63, 64].

Based on analysis of the *cox*1 DNA barcode region, sequences identified as *C. obsoletus* group into two major phylogenetic clades (Figs. 2, 3). All 67 sequences of *C. obsoletus* specimens obtained in the study conformed to one of these two clade (Clade 1) alongside sequences obtained during previous UK-based studies [25]. Clade 1 is thought to represent the classical morphological description of *C. obsoletus* [24] while the second clade includes specimens which have previously been associated with the ‘dark’ form of *C. obsoletus* in continental Europe [28]. Clade 2a (Figs. 2, 3) groups sequences of specimens which have been previously referenced as *C. obsoletus* O2 [59], while Clade 2b (Figs. 2, 3) contains a mixture of specimens which have been previously referenced as *C. obsoletus* O1, O2 and O3 [65]. In contrast, *C. scoticus, C. chiopterus* and *C. dewulfi* were all strongly supported as monophyletic clades with relatively low intraspecific sequence differences (Figs. 2, 4, 5, 6, Table 4).

Within the UK, further assessment of cryptic species prevalence and additional multi-locus studies are required across a wide-geographic area, both to assess the robustness of current PCR multiplex assays [25, 66] used to determine species in studies and to contribute to understanding relationships between phylogenetics and vector competence for arboviruses within the subgenus *Avaritia*. At a regional scale, studies are required that link both northwestern and northeastern European fauna and Nearctic species of the subgenus *Avaritia*. Several species in this group remain entirely uncharacterised using molecular markers, e.g. *C. sanguisuga* (Coquillett). Such data will then make decisions whether the observed sequence diversity represents novel species or potentially species that require resurrection from synonymy permissible, e.g. *C. dobyi* Callot & Kremer [67]. To resolve these issues it is particularly important that specimens are collected from the species type-locality in order to confirm that subsequent specimens are in fact conspecific (see Harrup et al. [68] for review).

Inaccurate species identification can have significant impacts on epidemiological investigations and control attempts when species have divergent vector competences and/or habitat and host preferences. Examination of the relative relationship between species within the subgenus *Avaritia* and confirmation of the monophyletic status of the subgenus, coupled with further arbovirus vector competence experiments will allow investigation of whether arbovirus vector competence is an ancestral character in the Obsoletus complex as previously proposed for the Imicola complex [69] or whether vector competence has evolved multiple times within this group.

## Conclusions

Methods described in this study provide the means to blood-feed Palaearctic *Culicoides* for vector competence studies and colonisation attempts. Susceptibility to SBV infection was 7.3% for membrane-fed members of the subgenus *Avaritia* and 1.1% for pledglet-fed. Both *C. obsoletus* and *C. scoticus* were confirmed as being susceptible to infection with SBV, with potential evidence of cryptic species within UK Obsoletus complex specimens; however the implications of cryptic diversity in the Obsoletus complex on arbovirus transmission remains unknown. This is important as these species are the most abundant *Culicoides* biting midges on farms across northwestern Europe and arboviruses transmitted by them continue to emerge in this region.

## Additional files

Additional file 1: Table S1. GenBank sequences used in the genetic analyses of Obsoletus complex of *Culicoides*. Includes references listed for GenBank sequences. (DOCX 79 kb)

Additional file 2: Table S2. Blood-feeding data for UK *Culicoides* exposed to membrane types**.** Number of *Culicoides* of each physiological state recorded following exposure to blood-feeding apparatus. (XLSX 21 kb)

## Abbreviations

AIC: Akaike information criterion
BIC: Bayesian information criterion
BIN: Barcode index number
BTV: Bluetongue virus
DNA: Deoxyribonucleic acid
HKY+G: Hasegawa-Kishino-Yano with gamma-distribution rates
*cox1*: Mitochondrial cytochrome *c* oxidase subunit 1 gene
OVI: Onderstepoort Veterinary Institute
RDF: Roehl haplotype data files
RH: Relative humidity
RNA: Ribonucleic acid
RT-qPCR: Quantitative reverse transcription polymerase chain reaction
SBV: Schmallenberg virus
