# Supplementary material for: Blood-feeding, susceptibility to infection with Schmallenberg virus and phylogenetics of Culicoides (Diptera: Ceratopogonidae) from the United Kingdom
Source: Parasit Vectors. 2018 Feb 27;11:116. doi: 10.1186/s13071-018-2650-x (PMC6389053; doi:10.1186/s13071-018-2650-x)
Supplement: Supplementary file 1 — GenBank sequences used in the genetic analyses of Obsoletus complex of Culicoides. Includes references listed for GenBank sequences. (DOCX 79 kb) [file 13071_2018_2650_MOESM1_ESM.docx]

**Additional file**

**Table S1. GenBank sequences used in the genetic analyses of Obsoletus group *Culicoides*.**

| **Species** | **Accession number** | **Reference** |
| --- | --- | --- |
| ***C. chiopterus* (Meigen), 1830** | AM236747-AM236751 | [[1](#_ENREF_1)] |
|  | HQ824397-HQ824412 | [[2](#_ENREF_2)] |
|  | JQ620044-JQ620048 | [[3](#_ENREF_3)] |
|  | JQ683259-JQ683262 | [[4](#_ENREF_4)] |
|  | JQ978440 | [[5](#_ENREF_5)] |
|  | KF802219-KF802237 | [[6](#_ENREF_6)] |
|  | KJ162976 | [[7](#_ENREF_7)] |
|  | KJ624070 | [[8](#_ENREF_8)] |
|  | KM940185; KM941505 | Submitted, unpublished: Biodiversity Instutute of Ontario, Canada |
| ***C. dewulfi* Goetghebuer, 1936** | AM236672-AM236707 | [[1](#_ENREF_1)] |
|  | HM022876-HM022881 | [[6](#_ENREF_6), [9](#_ENREF_9)] |
|  | HQ824413-HQ824416 | [[2](#_ENREF_2)] |
|  | JQ620063-JQ620067 | [[3](#_ENREF_3)] |
|  | JQ683263 | [[4](#_ENREF_4)] |
|  | JQ978428 | [[5](#_ENREF_5)] |
|  | KF802203-KF802218 | [[6](#_ENREF_6)] |
|  | KJ162977 | [[7](#_ENREF_7)] |
|  | KJ624076 | [[8](#_ENREF_8)] |
| ***C. obsoletus* (Meigen), 1818** | AM236652-AM236671 | [[1](#_ENREF_1)] |
|  | DQ162808-DQ162816 | [[10](#_ENREF_10)] |
|  | HM022792-HM022856 | [[6](#_ENREF_6), [9](#_ENREF_9)] |
|  | HQ824371-HQ824384 | [[2](#_ENREF_2)] |
|  | JQ620130-JQ620144 | [[3](#_ENREF_3)] |
|  | JQ683284-JQ683295 | [[4](#_ENREF_4)] |
|  | JQ740594-JQ740596 | [[11](#_ENREF_11)] |
|  | JQ978426; JQ978438; JQ978448; JQ978451; JQ978454; JQ978459; JQ978461- JQ978463; JQ978465- JQ978466 | [[5](#_ENREF_5)] |
|  | KJ162996 | [[7](#_ENREF_7)] |
|  | KJ624103-KJ624105 | [[8](#_ENREF_8)] |
|  | HM412931; HM551675; HQ982136; JF867960; JF868192; JN290741; KM632348; KM639363; KM899171; KM904109; KM951115; KM952825; KM960097; KM960328; KM961043; KM989870; KM990008; KR524802; KR640325; KR712308; KR713482; KR721032; KR725804; KR726264; KR727378; KR729918; KR740439; KR954151; KT075883; KT077512; KT079433; KT079608; KT079636; KT079766; KT080423; KT081567; KT082647; KT083854; KT084166; KT085035; KT087094; KT087368; KT088204; KT089881; KT091511; KT092811; KT094581; KT095358; KT095367; KT096571; KT112187 | Submitted, unpublished: Biodiversity Instutute of Ontario, Canada |
| ***C. scoticus* Downes and Kettle, 1952** | AM236625-AM236651 | [[1](#_ENREF_1)] |
|  | DQ162804-DQ162807 | [[10](#_ENREF_10)] |
|  | HM022857-HM022875 | [[6](#_ENREF_6), [9](#_ENREF_9)] |
|  | HQ824385-HQ824395 | [[2](#_ENREF_2)] |
|  | JQ620202-JQ620210 | [[3](#_ENREF_3)] |
|  | JQ683353-JQ683361 | [[4](#_ENREF_4)] |
|  | JQ978417- JQ978425; JQ978427; JQ978429- JQ978432; JQ978458; JQ978460 | [[5](#_ENREF_5)] |
|  | KJ624126 | [[8](#_ENREF_8)] |

Figure S1. Pairwise genetic distances (uncorrected percent sequence distances) across all species in the study. Box and whisker plots (left) and f**requency distribution plot (right) of** interspecific [closest non-conspecific (yellow)] and intraspecific [the furthest intraspecific distance among its own species (red)] pairwise genetic distances (uncorrected percent sequence distances) across all species in study. Areas where the intra- and inter-specific distances overlap shown in orange.


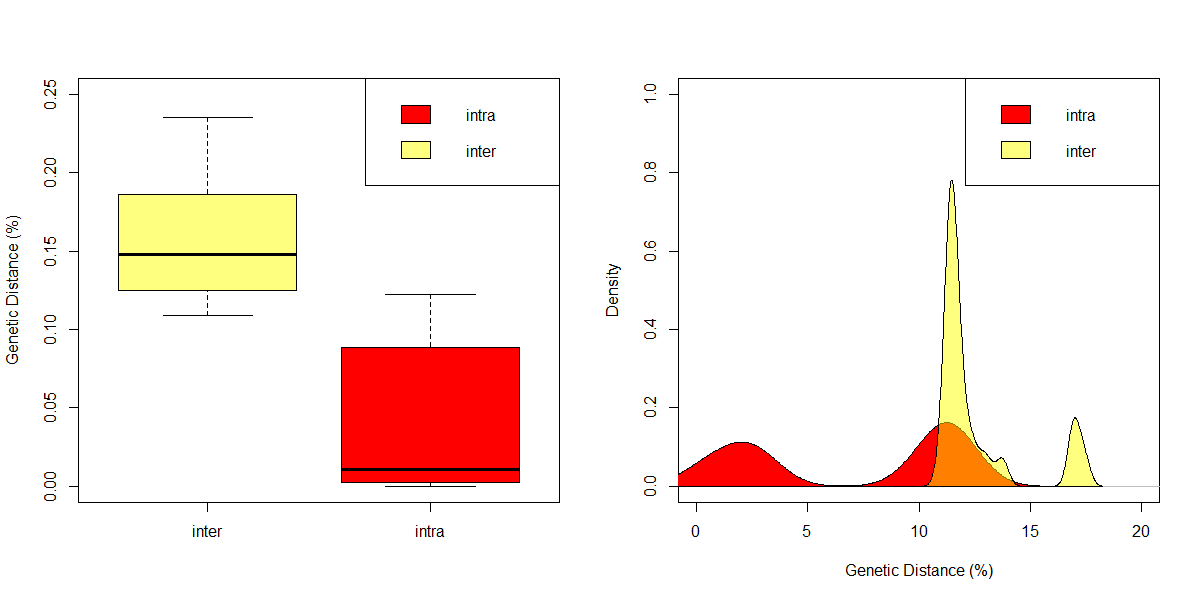


****Figure S2.**** Pairwise genetic distances (uncorrected percent sequence distances) by species. Box and whisker plots of the interspecific [closest non-conspecific (yellow)] and intraspecific [the furthest intraspecific distance among its own species (red)] pairwise genetic distances (uncorrected percent sequence distances) by species. Areas where the intra- and inter-specific distances overlap shown in orange.

*
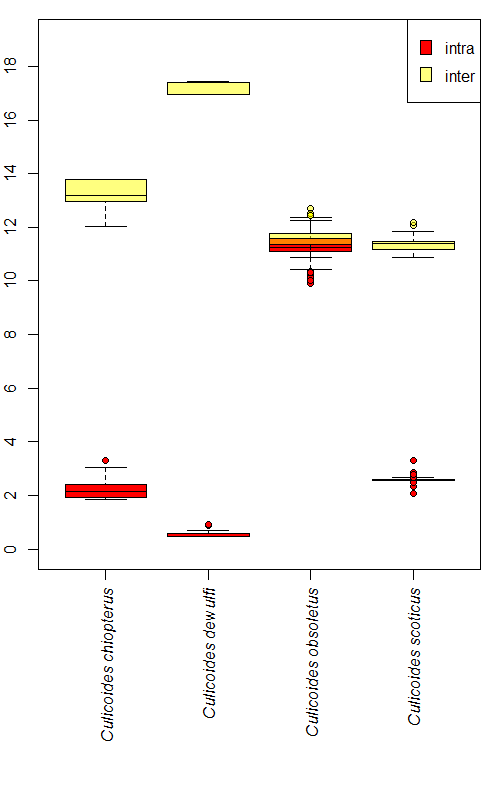
*

**References**

1. Nolan DV, Carpenter S, Barber J, Mellor PS, Dallas JF, Mordue Luntz AJ, Piertney SB: **Rapid diagnostic PCR assays for members of the *Culicoides obsoletus* and *Culicoides pulicaris* species complexes, implicated vectors of bluetongue virus in Europe**. *Vet Microbiol* 2007, **124**(1-2):82-94.

2. Wenk CE, Kaufmann C, Schaffner F, Mathis A: **Molecular characterization of Swiss Ceratopogonidae (Diptera) and evaluation of real-time PCR assays for the identification of *Culicoides* biting midges**. *Vet Parasitol* 2012, **184**(2-4):258-266.

3. Ander M, Troell K, Chirico J: **Barcoding of biting midges in the genus *Culicoides*: a tool for species determination**. *Med Vet Entomol* 2013, **27**(3):323-331.

4. Lassen SB, Nielsen SA, Kristensen M: **Identity and diversity of blood meal hosts of biting midges (Diptera: ceratopogonidae: *Culicoides* latreille) in Denmark**. *Parasite Vector* 2012, **5**:143.

5. Pettersson E, Bensch S, Ander M, Chirico J, Sigvald R, Ignell R: **Molecular identification of bloodmeals and species composition in *Culicoides* biting midges**. *Med Vet Entomol* 2013, **27**(1):104-112.

6. Henni LH, Sauvage F, Ninio C, Depaquit J, Augot D: **Wing geometry as a tool for discrimination of Obsoletus group (Diptera: Ceratopogonidae: Culicoides) in France**. *Infect Genet Evol* 2014, **21**:110-117.

7. Bellis G, Dyce A, Gopurenko D, Yanase T, Garros C, Labuschagne K, Mitchell A: **Revision of the *Culicoides* Avaritia *Imicola* complex Khamala & Kettle (Diptera: Ceratopogonidae) from the Australasian region**. *Zootaxa* 2014, **3768**(4):401.

8. Sarašová A, Kočišová A, Halán M, Delécolle JC, Mathieu B: **Morphological and molecular analysis of the genus *Culicoides* (Diptera: Ceratopogonidae) in Slovakia with five new records**. *Zootaxa* 2014, **3872**(5):541-560.

9. Augot D, Sauvage F, Jouet D, Simphal E, Veuille M, Couloux A, Kaltenbach ML, Depaquit J: **Discrimination of *Culicoides obsoletus* and *Culicoides scoticus*, potential bluetongue vectors, by morphometrical and mitochondrial cytochrome oxidase subunit I analysis**. *Infect Genet Evol* 2010, **10**(5):629-637.

10. Pagès N, Sarto i Monteys V: **Differentiation of *Culicoides obsoletus* and *Culicoides scoticus* (Diptera: Ceratopogonidae) based on Mitochondrial cytochrome oxidase subunit I**. *J Med Entomol* 2005, **42**:1026-1034.

11. Martinez-de la Puente J, Martinez J, Ferraguti M, Morales-de la Nuez A, Castro N, Figuerola J: **Genetic characterization and molecular identification of the bloodmeal sources of the potential bluetongue vector *Culicoides obsoletus* in the Canary Islands, Spain**. *Parasite Vector* 2012, **5**:147.
